# Supplementary material for: Exercise-Induced Bronchoconstriction in Children: State of the Art from Diagnosis to Treatment
Source: J Clin Med. 2024 Aug 5;13(15):4558. doi: 10.3390/jcm13154558 (PMC11312884; doi:10.3390/jcm13154558)
Supplement: Supplementary file 1 [file jcm-13-04558-s001.zip › jcm-3081097-supplementary.pdf]

Supplementary material

Critical analysis of key studies

Epidemiology and risk factors			
**Study (first author, year, [Reference])**	**Type of study**	**Strengths**	**Limitations/Methodological concerns**
Caillaud, 2014 [1]	Population-based study	Large population-based sample (n=7,781 schoolchildren); use of objective tool to assess exercise induced bronchoconstriction (EIB) and sensitization	Only urban schoolchildren<br>High variability influenced by demographic and geographical factors
Jones, 1996 [2]	Population-based study	Large sample (n=1,137 asthmatic children); first analysis on multiple ethnicities	Unselected sample<br>High variability influenced by demographic and geographical factors
Rodriguez, 2020 [3]	Systematic review and meta-analysis	Numbers of study analysed (n=60); effects of sex and sport on EIB	No paediatric data, post-pubertal athletes<br>Differences in protocol used in assessing EIB
Pathogenesis			
**Study (first author, year, [Reference])**	**Type of study**	**Strengths**	**Limitations/Methodological concerns**
Bonini, 2015 [4]	Narrative review	Comprehensive report and update of literature	Type of article<br>No time interval of research
Stelmach, 2016 [5]	Cohort study	Study of environmental factors, large sample (n=1,370 school-children)	Only urban schoolchildren; environmental tobacco not taken into account<br>Study sample was not adequate to allow full sub-group analysis
Baek, 2013 [6]	RCT	Assessing the levels of leukotriene E(4) in patients	Small sample size (n=24 patients enrolled)

		treated with montelukast or fluticasone	Exclusion of patients with unstable asthma
Diagnosis, Differential diagnosis			
**Study (first author, year, [Reference])**	**Type of study**	**Strengths**	**Limitations/Methodological concerns**
Parsons, 2013 [7]	Clinical practice guideline, American Thoracic Society	Comprehensive evidence-based guidelines, expert panel, GRADE approach	Lack of data from publication onwards
Hallstrand, 2018 [8]	Document on technical standards of Bronchial Challenge Test	International ERS and ATS task force, 370 documents analysed	Focused on Bronchial Challenge testing, no other test analysed<br>Lack of data from publication onwards
Van Brussel, 2019 [9]	Review on technical standards and interpretation of CPET	Based on 15-years experience and recent literature and technical standards	Based on a single-centre experience<br>Lack of data from publication onwards
Weiss, 2009	Narrative review	Comprehensive report and update of literature on differential diagnosis of EIB	Type of article<br>No time interval of research
**Treatment**			
**Study (first author, year, [Reference])**	**Type of study**	**Strengths**	**Limitations/Methodological concerns**
Parsons, 2013 [7]	Clinical practice guideline, American Thoracic Society	Comprehensive evidence-based guidelines, expert panel, GRADE approach	Lack of data from publication onwards
Bonini, 2013 [10]	Cochrane Database systematic review	Only RCT included	Lack of data from publication onwards
Backer, 2018 [11]	Narrative review	Comprehensive report and update of literature	Type of article<br>No time interval of research

Hengeveld, 2022 [12]	Prospective cohort-study	Assessment of efficacy of a single-dose of ICS vs a 4-week treatment	Small sample size (n=32)
Follow-up			
**Study (first author, year, [Reference])**	**Type of study**	**Strengths**	**Limitations/Methodological concerns**
Parsons, 2013 [7]	Clinical practice guideline, American Thoracic Society	Comprehensive evidence-based guidelines, expert panel, GRADE approach	Lack of data from publication onwards
Johansson, 2019 [13]	Prospective cohort study	Large sample (n=2,309), wide-range questionnaire	Self-reported data<br>No assessment on severity of EIB

References

1. Caillaud, D.; Horo, K.; Baiz, N.; Banerjee, S.; Charpin, D.; Lavaud, F.; de Blay F.; Raherison C.; Annesi-Maesano I. Exercise-induced bronchospasm related to different phenotypes of rhinitis without asthma in primary schoolchildren: The French Six Cities Study. *Clin. Exp. Allergy.* **2014**, *44*, 858–866.
2. Jones, C.O.; Qureshi, S.; Rona, R.J.; Chinn, S. Exercise-induced bronchoconstriction by ethnicity and presence of asthma in British nine year olds. *Thorax* **1996**, *51*, 1134–1136.
3. Rodriguez Bauza, D.E.; Silveyra, P. Sex Differences in Exercise-Induced Bronchoconstriction in Athletes: A Systematic Review and Meta-Analysis. *Int. J. Environ. Res. Public Health* **2020**, *17*, 7270.
4. Bonini, M.; Gramiccioni, C.; Fioretti, D.; Ruckert, B.; Rinaldi, M.; Akdis, C.; Todaro A.; Palange P.; Carlsen K.H.; Pelliccia A.; et al. Asthma, allergy and the Olympics: A 12-year survey in elite athletes. *Curr. Opin. Allergy Clin. Immunol.* **2015**, *15*, 184.
5. Stelmach, I.; Cichalewski, Ł.; Majak, P.; Smejda, K.; Podlecka, D.; Jerzyńska, J.; Lavaud F.; Taytard A.; Annesi-maesano I. School environmental factors are predictive for exercise-induced symptoms in children. *Respir. Med.* **2016**, *112*, 25–30.

6. Baek, H.S.; Cho, J.; Kim, J.H.; Oh, J.W.; Lee, H.B. Ratio of leukotriene e(4) to exhaled nitric oxide and the therapeutic response in children with exercise-induced bronchoconstriction. *Allergy Asthma Immunol. Res.* **2013**, *5*, 26–33.
7. Parsons, J.P.; Hallstrand, T.S.; Mastronarde, J.G.; Kaminsky, D.A.; Rundell, K.W.; Hull, J.H.; Storms W.W.; Weiler J.M.; Cheek F.M.; Wilson K.C.; et al. An official American Thoracic Society clinical practice guideline: Exercise-induced bronchoconstriction. *Am. J. Respir. Crit. Care Med.* **2013**, *187*, 1016–1027.
8. Hallstrand, T.S.; Leuppi, J.D.; Joos, G.; Hall, G.L.; Carlsen, K.-H.; Kaminsky, D.A.; Coates A.L.; Cockcroft D.W.; Culver B.H.; Diamant Z.; et al. ERS technical standard on bronchial challenge testing: Pathophysiology and methodology of indirect airway challenge testing. *Eur. Respir. J.* **2018**, *52*, 1801033.
9. Van Brussel, M.; Bongers, B.C.; Hulzebos, E.H.J.; Burghard, M.; Takken, T. A Systematic Approach to Interpreting the Cardiopulmonary Exercise Test in Pediatrics. *Pediatr. Exerc. Sci.* **2019**, *31*, 194–203.
10. Bonini, M.; Di Mambro, C.; Calderon, M.A.; Compalati, E.; Schünemann, H.; Durham, S.; Canonica G.W. Beta$_2$-agonists for exercise-induced asthma. *Cochrane Database Syst. Rev.* **2013**, CD003564.
11. Backer, V.; Mastronarde, J. Pharmacologic Strategies for Exercise-Induced Bronchospasm with a Focus on Athletes. *Immunol. Allergy Clin. N. Am.* **2018**, *38*, 231–243.
12. Hengeveld, V.S.; Lammers, N.; van der Kamp, M.R.; van der Palen, J.; Thio, B.J. Can the response to a single dose of beclomethasone dipropionate predict the outcome of long-term treatment in childhood exercise-induced bronchoconstriction? *Pediatr. Allergy Immunol.* **2022**, *33*, e13808.
13. Johansson, H.; Norlander, K.; Malinovschi, A. Increased prevalence of exercise-induced airway symptoms—A five-year follow-up from adolescence to young adulthood. *Respir. Med.* **2019**, *154*, 76–81.
